# Deformation and Failure Mechanism of Particulate Filled and Short Fiber Reinforced Thermoplastics: Detection and Analysis by Acoustic Emission Testing

**DOI:** 10.3390/polym13223931

**Published:** 2021-11-14

**Authors:** Milán Ferdinánd, Róbert Várdai, János Móczó, Béla Pukánszky

**Affiliations:** 1 Institute of Materials and Environmental Chemistry, Research Centre for Natural Sciences, P.O. Box 286, H-1519 Budapest, Hungary; vardai.robert@vbk.bme.hu (R.V.); jmoczo@edu.bme.hu (J.M.); pukanszky.bela@vbk.bme.hu (B.P.); 2 Laboratory of Plastics and Rubber Technology, Department of Physical Chemistry and Materials Science, Budapest University of Technology and Economics, P.O. Box 91, H-1521 Budapest, Hungary

**Keywords:** acoustic emission testing, local processes, polymer composite, deformation, failure, debonding, fiber pull out, fiber fracture, interfacial adhesion

## Abstract

Acoustic emission, the detection of signals during deformation, is a frequently used method for the study of local deformation processes occurring in heterogeneous polymer systems. Most of these processes result in the evolution of elastic waves which can be detected by appropriate sensors. The analysis of several parameters characterizing the waves offers valuable information about the possible deformation mechanism. The acoustic emission testing of composites may yield very different number of signals from a few hundred to more than 100,000. This latter was proved to be affected mainly by particle size, interfacial adhesion and composition, but other factors, such as matrix modulus and specimen size, also influence it. Local deformation processes are claimed to have a strong effect on macroscopic properties. Indeed, a close correlation was found between the initiation stress of the dominating particle related process derived from acoustic emission testing and the tensile strength in both polypropylene (PP) and poly(lactic acid) (PLA) composites. However, in polyamide (PA)-based heterogeneous polymer systems, deformations related to the matrix dominated composite properties. Besides forecasting failure, the method makes possible the determination of the inherent strength of lignocellulosic fibers being around 40 MPa as well as the quantitative estimation of adhesion strength for composites in which interactions are created by mechanisms other than secondary forces. The proposed approach based on acoustic emission testing proved that in PP/CaCO_3_ composites, the strength of adhesion can be increased by ten times from about 100 mJ/m^2^ to almost 1000 mJ/m^2^ in the presence of a functionalized polymer.

## 1. Introduction

Polypropylene (PP) is one of the commodity polymers used in the largest quantities in various application areas due to its light weight, balanced performance/price ratio and easy recyclability as well. Extensively used homopolymer grades have a sufficiently large stiffness of approximately 1.5 GPa and acceptable strength; however, their impact strength is small, around 2 kJ/m^2^. PP is applied in several fields of the industry, e.g., in the automotive, construction and packaging sector and it is frequently used for the production of household appliances as well [1,2]. Among engineering thermoplastics, polyamide (PA) is a preferred material, especially in the automotive industry. PA possesses an exceptionally good combination of stiffness, strength and impact strength, while its heat resistance exceeds that of PP, which allows its use in under the hood applications [3,4]. Due to the increasing environmental awareness of the population polymers produced from renewable raw materials have gained considerable attention both by academia and industry. Among bioplastics, poly(lactic acid) (PLA) has been in the focus of intensive research recently, since it is produced from renewable agricultural raw materials, e.g., corn starch, and it has some advantageous properties. PLA is a relatively stiff polymer, with a stiffness of 3 GPa, while its tensile strength can exceed 60 MPa. However, the polymer is very brittle and it is sensitive to water during processing. PLA is applied as food packaging material, agricultural film and disposable plastic bag, as it is compostable [5,6].

The above-mentioned polymers are also widely used as structural parts in load-bearing applications. Materials applied under these conditions are required to have sufficiently large stiffness, strength, dimensional stability and heat deflection temperature as well. Toughness is also a frequent requirement from parts exposed to dynamic load. Since neat polymers usually do not satisfy the high technical requirements listed above, modification is often needed, i.e., a second component has to be added to the polymer. Blending with elastomers is the traditional approach to increase impact resistance [7,8]. Particulate filled polymers offer large stiffness and dimensional stability [9], while short fiber-reinforced composites, with either synthetic or natural reinforcement, achieve a beneficial combination of large stiffness and strength [10,11]. Since the traditional modification routes, i.e., the addition of elastomer or filler/fiber, usually alter only one targeted property in the desired direction, multicomponent materials containing at least two different additives are also prepared in order to reach a balanced property profile [12,13].

At first glance, modified polymers seem to differ significantly from each other, but they are all materials with heterogeneous structure. Under the effect of external load heterogeneities induce stress concentration, the magnitude of which depends on the geometry of the inclusions, the elastic properties of the components and interfacial adhesion [14]. Heterogeneous stress distribution and local stress maxima initiate local micromechanical deformation processes which strongly influence failure behavior, thus the overall performance of heterogeneous polymer systems. Both matrix and particle related processes can take place during the deformation of these materials. A certain extent of shear yielding, i.e., the slipping of larger structural units of the matrix, always occurs both in crystalline and amorphous polymers [15]. In elastomer-modified polymers [16], in PA and PLA [17], cavitation may also take place resulting in the formation of voids. Crazing was also observed in numerous polymers including polystyrene (PS) and PP [15,18]. Debonding, i.e., the separation of the interface between the matrix and the filler was proved to be the dominating deformation mechanism both in particulate filled and in some short fiber reinforced composites [19,20,21]. Besides debonding, several other processes, such as the pull out, fracture and buckling of the fibers may occur in the latter as well [11,22]. Micromechanical deformations are competitive processes that can take place both consecutively and/or simultaneously. One of them usually dominates failure but the effect of the others on composite properties cannot be neglected either.

Taking into consideration their importance, the above-mentioned processes must be detected and studied in an appropriate way. Volume strain measurements (VOLS) can follow changes in the sample volume continuously during deformation [23,24]. Apart from shear yielding, most micromechanical deformation mechanisms are accompanied by volume increase, thus the technique cannot distinguish between them. Another approach frequently used for the analysis of local processes is acoustic emission (AE), the detection of elastic waves emitted during the elongation of the specimen [25]. Most local deformations emit sound [11,13,19,22] which can be detected by appropriate sensors. The characteristics of the elastic waves are closely related to the process emitting them, thus the analysis of the various parameters of the waves offers valuable information about the possible mechanism of the process. The approach is frequently used for the study of the deformation mechanism of fiber-reinforced composites [26,27]. Fiber related processes, especially the fracture of fibers is of sufficiently high energy, thus the detection of acoustic signals does not create any problem. However, in particulate-filled polymers and polymer blends the energy of the signals is much smaller, thus occasionally signals cannot be detected during deformation.

Although acoustic emission is more and more often used for the measurement and analysis of local deformation processes, based on our knowledge, a comprehensive study has not been published on its use for particulate and short fiber-reinforced composites yet. Consequently, besides presenting the principles of the method, our aim was to explore the most important factors determining acoustic activity, i.e., the number of signals developing during the measurement. We also wanted to review the processes which can be followed by the technique, present some examples for its application, and highlight the advantages and drawbacks of the approach. Practical relevance is also discussed in the final section of the paper.

## 2. Experimental

### 2.1. Materials

Four PP grades were used for sample preparation. The Tipplen H 543 F grade homopolymer (hPP1) with an MFR of 4 g/10 min, the Tipplen H 649 FH grade homopolymer (hPP2) with an MFR of 2.5 g/10 min and the Tipplen R 605 grade random copolymer (rPP) with an MFR of 2 g/10 min were supplied by the MOL Group, Tiszaújváros, Hungary. The Daplen HJ 325 MO grade homopolymer PP (hPP3) with an MFR of 50 g/10 min was acquired from Borealis GmbH, Linz, Austria. An ethylene-methacrylic acid copolymer partially neutralized by zinc hydroxide (ionomer) from DuPont, Wilmington, DE, USA was also applied in the experiments. The Bretax SRO2 grade lignosulfonate with sodium counter ions obtained from the Burgo Group SpA, Altavilla Vicentina, Italy was added to the ionomer in the study. The Ingeo 4032 D grade PLA with an MFR of 3.9 g/10 min was the product of NatureWorks, Minnetonka, MN, USA. The Domamide 27 PA6 with an MFR of 4 g/10 min was also investigated in the experiments, which was provided by Domo Chemicals, Premnitz, Germany. The MFR values of all polymers were determined at 230 °C and 2.16 kg. 

Various fillers and fibers were used in the study. Spheriglass grade glass beads from Potters Industry, Malvern, PA, USA had particle sizes covering a range from 10 to 203 μm average size. Five grades were used altogether. Glass beads were added to the polymer as received, while an aminosilane [(3-aminopropyl)triethoxysilane] coating was used to increase the strength of interfacial adhesion. Four Omyacarb grades of CaCO_3_, including Omyacarb 2GU, 15GU, 40GU and 130GU (Omya, Oftringen, Switzerland) were also used to modify PP. The average particle size of the fillers was 3.6, 21, 88 and 258 μm, respectively. Stearic acid coating was used to decrease, while the Orevac CA 100 grade maleated PP (MAPP1) purchased from Arkema, Colombes, France was applied to increase adhesion strength. The maleic-anhydride content of the functionalized polymer was 1 wt%, while its MFR was 112 g/10 min at 190 °C, 2.16 kg. Five different lignocellulosic fibers were also applied in the study, four wood flours and ground corn cob. Three of the wood flours, Arbocel CW 630 (volume average particle size, D[4,3] = 39.6 μm, aspect ratio, AR = 3.5), Filtracel EFC 1000 (D[4,3] = 213.1 μm, AR = 6.8), and Arbocel FT 400 (D[4,3] = 171 μm, AR = 12.6), were supplied by Rettenmaier and Söhne GmbH, Rosenberg, Germany, while the fourth, Lasole 200/150 (D[4,3] = 280.8 μm, AR = 5.4) was acquired from La.So.Le. EST Srl., Percoto, Italy. The heavy fraction of corn cob (D[4,3] = 143.4 μm, AR = 2.3) was the product of Bóly Zrt., Bóly, Hungary. Abbreviations are used for the above listed lignocellulosic fibers. The letters indicate origin of the fibers (wood flour, W, corn cob, CC), while numbers denote ten times their aspect ratio, e.g., wood flour with an aspect ratio of 12.6 is referred to as W126. Glass, poly(vinyl alcohol) (PVA) as well as sugarpalm fibers were also applied as reinforcement in the experiments. The ThermoFlow 636 grade glass fibers with a silane sizing were purchased from Johns Manville, Denver, CO, USA. The fibers had an initial diameter of 13 μm and a length of 4 mm. The PVA fiber was the Kuralon VPB-103 grade obtained from Kuraray, Tokyo, Japan. The original length of the fibers was 3 mm and their diameter 11 μm. The sugarpalm fibers were obtained as bundles from a local producer in Sidoarjo, Indonesia and were cut before homogenization. The average length of the fibers was 1 mm, while their diameter 150 μm. The Scona 6102 grade maleated PP (MAPP2) supplied by Byk-Chemie GmbH, Wesel, Germany was applied to increase the strength of interfacial adhesion in composites containing the above listed fibers. The functionalized polymer had a maleic-anhydride content of >0.9 wt% and an MFR of 25 g/10 min at 230 °C, 2.16 kg. The composition of all composites prepared in the study is summarized in Table 1.

### 2.2. Sample Preparation

Polymers and fillers/fibers were dried under vacuum at 105 °C for 4 h before processing. Components were homogenized using a Brabender DSK 42/7 twin-screw compounder (Brabender GmbH, Duisburg, Germany) at different set temperatures depending on the type of matrix polymer and at 40 rpm rate. Extrusion was repeated once in order to increase homogeneity. The granuled composites were dried at 80 °C for 4 h in an air-circulating oven, then, they were injection-molded into standard (ISO 527 1A) tensile bars of 4 mm thickness using a Demag IntElect 50/330–100 machine (Sumitomo Demag GmbH, Schwaig, Germany). Processing parameters were 50 bar back pressure, 40 mm/s injection speed, 25 s holding time, and 25 s cooling time. Processing temperatures varied depending on the matrix polymer. The temperature of the mold was set to 40 °C. The specimens were stored at ambient temperature (23 °C, 50% RH) for a week before further testing.

### 2.3. Characterization, Measurements

Tensile tests were carried out using an Instron 5566 universal testing machine (Instron, Norwood, MA, USA) with a gauge length of 115 mm and 5 mm/min crosshead speed. Volume strain was determined by measuring the change in one lateral dimension of the specimen by a strain transducer. The same dimensional changes were assumed to occur in both lateral directions. Acoustic emission signals were recorded with a Sensophone AED 404 apparatus (Geréb és Társa Kft., Budapest, Hungary). A single a11 resonance detector with the resonance frequency of 150 kHz was attached to the center of the specimen. The threshold level of detection was set to 20 dB. Scanning electron micrographs (SEM) were recorded on fracture surfaces created during tensile testing using a Jeol JSM 6380 LA apparatus (Jeol Ltd., Tokyo, Japan). The broken surfaces were sputtered with gold before recording the micrographs.

## 3. Principles

**Figure 1:**
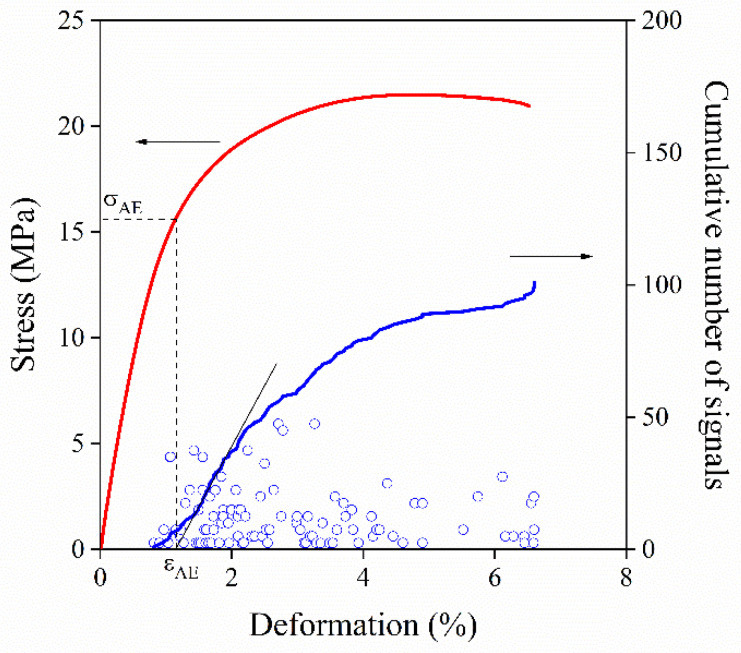
Results of the acoustic emission testing of a hPP1 composite containing 15 vol% CaCO_3_ with an average particle size of 3.6 μm. Solid lines are the cumulative number of signals trace (right axis) and the corresponding stress vs. deformation trace (left axis).

As mentioned earlier, under the effect of external load local deformation processes are initiated around inclusions in heterogeneous polymer systems, which can be studied by acoustic emission. The technique is based on the fact that if energy is suddenly released in a material, some of it dissipates in the form of elastic waves which can be detected by piezoelectric sensors. Voltage is induced in the sensor strained by these waves, thus data are stored as voltage vs. time functions during the measurement [28]. Basically, two types of AE signals can be distinguished: continuous and burst-like signals. While dislocation or grinding are accompanied by the development of continuous signals, the burst-like ones are emitted by sudden events, such as micromechanical deformations. Since individual acoustic signals are elastic waves, they can be characterized by several parameters: amplitude, counts, duration, frequency (counts/duration) and energy [29]. The amplitude and the frequency of the signals are frequently used for the identification of local deformation processes in fiber reinforced composites. It was shown earlier that debonding emits signals with relatively low amplitudes, while larger amplitudes can be assigned to fiber pull out or fracture [21,27]. A typical representation of the results of acoustic emission testing carried out on a PP/CaCO_3_ composite with an average particle size of 3.6 μm at 15 vol% filler content is demonstrated in Figure 1. Individual acoustic signals (events) are indicated as blue circles, while the corresponding stress vs. deformation trace is also plotted for comparison. Only a few signals were recorded during the deformation of the composite corroborating our previous statement that in particulate filled polymers both the number and the energy of the signals is relatively small, thus detection is difficult. Acoustic events appear only above a certain deformation and although the amplitude of the signals cover a wide range, the average value is still quite small (the vertical position of the acoustic event corresponds to its amplitude in this representation). It is worth noting that amplitude is plotted in a logarithmic unit, dB, since voltage values cover a range of several orders of magnitude. In order to facilitate evaluation and obtain further information, another quantity, the cumulative number of signals summarizing all the acoustic events up to a certain deformation is also plotted in Figure 1. One can see that the number of signals start to increase above ~1% deformation and the majority of the events are recorded before yield stress is reached. The demonstrated step-like S-shape curve is characteristic for debonding [19]. However, in a stiffer material with smaller deformability, the saturation-like behavior cannot be established, because the sample breaks and we do not reach the saturation value. Although CaCO_3_ particles certainly debond from the matrix, other local processes may also take place during the elongation of the specimen. PP deforms by shear yielding and occasionally cracks develop in the crystalline phase as well, but these processes do not emit much sound. As indicated in Figure 1, the plotting of the cumulative number of signals trace also allows us to determine a characteristic deformation $(\varepsilon_{AE})\;$and the corresponding stress value $\left(\sigma_{AE}\right)$ indicating the initiation of the dominating local process, debonding in this case. These values are characteristic for the process and they can be used for further evaluation and identification.

## 4. Factors

The acoustic emission testing of a composite may yield very different number of signals with widely differing characteristics. Some particulate filled composites occasionally do not give a signal at all, while glass fiber-reinforced composites were shown to emit more than 100,000 signals during their deformation [11]. In order to explore the most important factors determining the number of signals, we focused our attention on debonding, since it is the dominating micromechanical deformation process in particulate filled polymer composites and it is studied by a number of research groups [19,20,21,30]. Debonding stress is directly proportional to the strength of interfacial adhesion, while inversely to particle size. The initiation stress of debonding is affected also by thermal stresses and the Young’s modulus of the matrix [30]. Based on these facts, several assumptions can be made for expected changes in the acoustic emission behaviour of polymer composites. Debonding stress increases with decreasing particle size, thus we may assume that also the energy of debonding would increase, which may result in increased number of signals. Coupling resulting in increased adhesion should result in changes into the same direction, although very strong adhesion may prevent the debonding of the particles. On the other hand, decreased adhesion may lead to very easy debonding and to the loss of sound. Similarly, we may also assume that below a certain particle size debonding does not take place at all, thus acoustic emission signals will not be recorded at all. In order to verify our assumptions, acoustic emission measurements were carried out on a large number of particulate filled PP composites containing CaCO_3_ or glass beads with different particle sizes and surface modifications.

**Figure 2:**
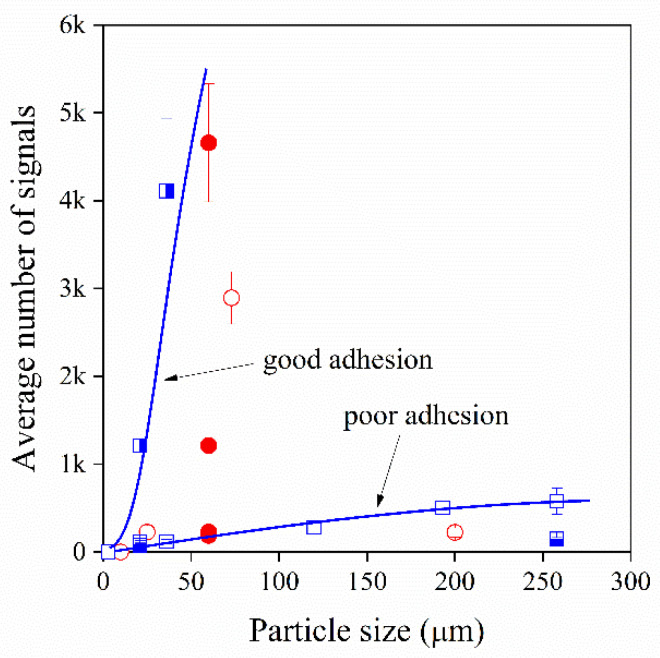
Effect of particle size and interfacial adhesion on the number of signals detected by acoustic emission in particulate filled hPP1 composites.

If the Young’s modulus of the matrix is kept constant, particle size and interfacial adhesion are in all probability the most important factors affecting acoustic activity. Figure 2 offers a good insight into the effect of these factors on the acoustic emission behaviour of the various composites prepared in the study. The average number of signals slightly increase with increasing particle size for both fillers but not in a simple manner. A threshold particle size seems to exist for sound development, since small particles below 10–20 μm of size do not emit a signal at all. Debonding stress might be larger than the yield stress or tensile strength of the composite, thus debonding cannot take place in this case. Although larger particles seem to give more signals during the measurement, glass beads with 200 μm size are not very active and the number of acoustic events detected is smaller again than at intermediate sizes. The value obtained for glass beads with an average particle size of 71 μm is larger than expected that needs further explanation. It is worth to note that values obtained for both glass beads and CaCO_3_ fillers fall onto the same correlation indicating similar strength of interfacial interactions in the two cases.

The effect of interfacial adhesion on acoustic activity is also demonstrated in Figure 2. Increased adhesion, i.e., the presence of MAPP, leads to the drastic increase in the number of signals detected, it increased from 60 to 1200 for the smaller and from 500 to more than 10,000 for the largest particles. On the other hand, the treatment of CaCO_3_ with stearic acid resulting in the decrease in the strength of interfacial adhesion between the filler and the matrix results in a smaller number of events. Obviously, the strength of interfacial interactions has an even stronger effect on the number of signals emitted during the measurement than particle size.

Composites containing the smallest particles do not give a signal at all, which might be explained by the absence of debonding. In order to confirm our explanation, volume strain measurements were carried out on the composites containing the smallest CaCO_3_ particles with the average size of 3.6 μm in the presence of MAPP. Although we refrain from presenting the results in order to keep the paper at a reasonable length, volume was observed to increase continuously and depended on filler content despite the small particle size and the strong interfacial adhesion. These observations indicate that at least a part of particles debonded from the matrix, but no signals were emitted in the process. Obviously, besides particle size and interfacial adhesion, another, a neglected factor also influences acoustic activity. We assume that this factor is the debonded volume. If a high-energy signal develops in a large volume, the piezoelectric sensor can pick it up, but if the volume is small, the signal is weak and/or it is attenuated during propagation, it does not reach the detector. This would explain the lack of sound for small particles with excellent adhesion to the matrix, since sound is generated in a very small volume in this case.

**Figure 3:**
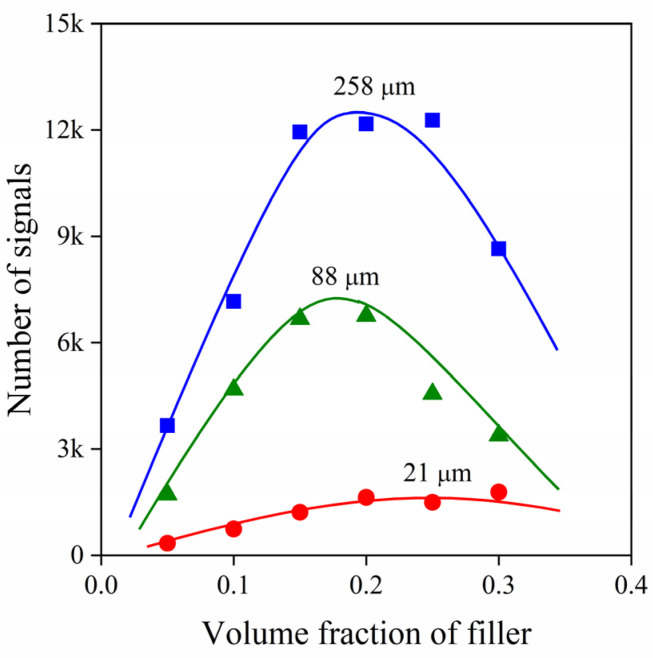
Effect of composition on the number of signals detected by acoustic emission in particulate filled hPP1 composites in the presence of MAPP1.

It seems obvious, but filler content also influences signal development as shown in Figure 3. Although in PP/CaCO_3_ composites the number of signals increase with increasing filler content irrespectively of the average particle size, a considerable drop occurs above a certain loading which needs explanation. At large filler content, the detector may not distinguish among simultaneously developing signals and the generated elastic waves interfere with each other or with the debonded volume thus resulting in their extinction and a smaller number of acoustic events.

Besides the factors mentioned above, matrix modulus and specimen size were shown to affect sound generation as well. The large extent of plastic deformation occurring in elastomers with low modulus hinder the propagation of elastic waves generated around heterogeneities which leads to a decrease in the number of signals recorded. The same explanation can be applied for specimens with small size. Elastic waves are attenuated due to the considerable plastic deformation taking place within the specimen which results in the diminution of the number of acoustic events.

**Figure 4:**
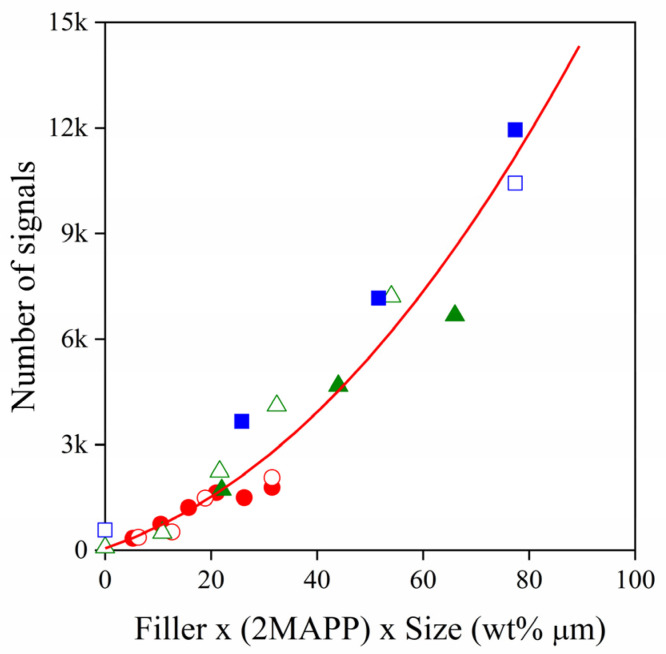
Correlation between the number of signals and the simple combination of composition, adhesion and particle size.

It was shown in this section that the generation as well as the propagation of sound waves in polymer composites are complicated processes influenced by several factors. Even so, the number of signals detected can be described quite well by the simple combination of three variables investigated, as it is demonstrated in Figure 4. The apparently good correlation indicates that filler content, interfacial adhesion and particle size do indeed strongly affect signal development.

## 5. Processes

**Figure 5:**
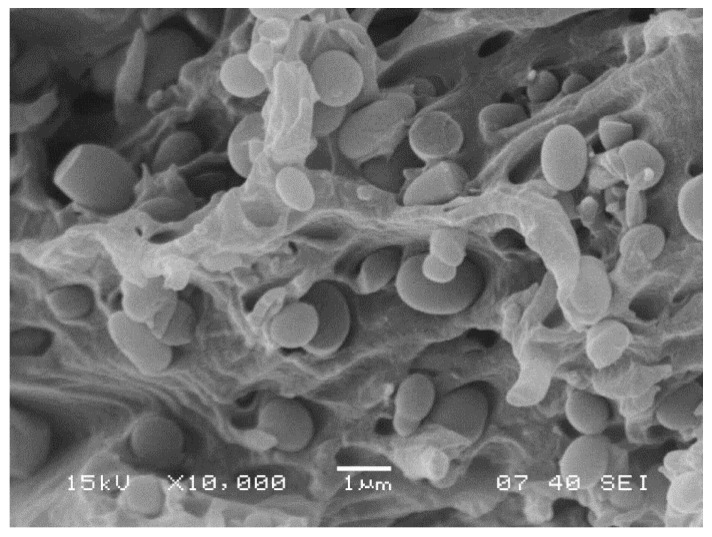
SEM micrograph recorded on the fracture surface of an ionomer/lignin blend. Lignin content: 20 vol%. Debonding of the particles.

Debonding is the dominating local deformation process in heterogeneous polymer systems containing particles as it was stated earlier. A SEM micrograph recorded on the fracture surface of an ionomomer/lignin blend containing 20 vol% lignin (Figure 5) provides unambiguous proof regarding the presence of the process. The clean surface of the small particles, the separated interfaces and the holes indicate the occurrence of debonding without any doubt.

**Figure 6:**
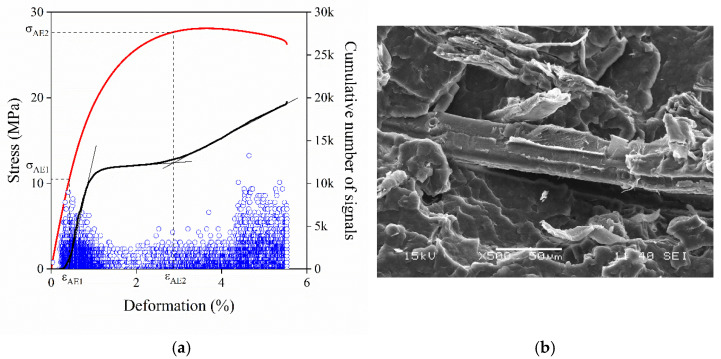
Consecutive local processes occurring in a hPP3 composite containing 15 vol% W68. Debonding and pull out of wood flour particles. (**a**) Result of the acoustic emission testing. Solid lines are the cumulative number of signals trace (right axis) and the corresponding stress vs. deformation trace (left axis). (**b**) SEM micrograph recorded on the fracture surface of the composite.

With increasing anisotropy of the reinforcement, besides debonding, other particle related processes can also take place during deformation. A good example is provided in Figure 6a, in which the results of the acoustic emission measurement carried out on a PP composite containing 15 vol% wood flour are presented. The two separate groups of individual signals as well as the two steps appearing on the cumulative number of signals trace indicate the occurrence of two consecutive local deformation processes with different mechanisms. Based on our previous experience [31], the first process might be debonding, since wood particles can easily separate from the matrix due to their large size. The process initiates at a small characteristic stress value and it is completed in a narrow deformation range. In all probability, the second process can be assigned to the pull out or the fracture of wood flour particles, which are initiated at a larger characteristic deformation and thus stress value. The process is accompanied by the emission of signals with somewhat larger amplitudes. Although acoustic emission testing proves the occurrence of two consecutive local deformation processes, these cannot be identified unambiguously based on the results. In order to support the conclusions drawn from the measurement and help further identification, SEM micrographs were recorded on the fracture surface of the composite. The separation of a large wood flour particle from the matrix as well as the pull out of the fiber can be clearly observed on the micrograph presented in Figure 6b, thus confirming our previous assumptions.

**Figure 7:**
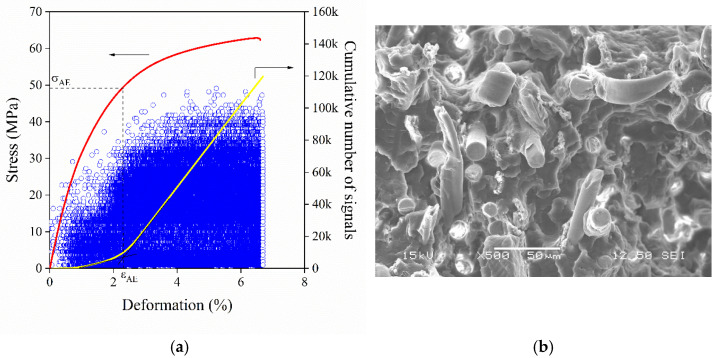
Simultaneous micromechanical deformations taking place in a hybrid hPP3 composite containing 15 vol% glass fiber and 15 vol% PVA fiber in the presence of MAPP2. Perpendicular fracture of both fibers. (**a**) Result of the acoustic emission measurement. (**b**) SEM micrograph recorded on the fracture surface of the composite.

If the fibers are long enough and the adhesion is good between them and the matrix, fibers predominantly break during the deformation of the composite. A representative cumulative number of signals trace for fiber fracture obtained on a hybrid PP composite containing 15 vol% glass fiber and 15 vol% PVA fiber in the presence of MAPP shows intensive fiber fracture in Figure 7a. The continuously increasing cumulative number of signals trace, the relatively large amplitudes and large characteristic stress value indicate the occurrence of fiber fracture. As it was mentioned earlier, the fracture of fibers is of sufficiently high energy, which leads to the development of more than 100,000 signals in this case. Fiber fracture is further supported by the SEM micrograph recorded on the fracture surface of the same composite (Figure 7b). Both glass and PVA fibers are coated with the matrix polymer indicating good adhesion. Improved adhesion results in effective stress transfer between the components leading to the fracture of the fibers perpendicularly to their axis. It is important to note that although both fibers break during deformation, only one step appears on the cumulative number of signals trace. This calls attention to the fact that acoustic emission cannot distinguish among simultaneously occurring local deformation processes, thus additional techniques are needed for evaluation.

**Figure 8:**
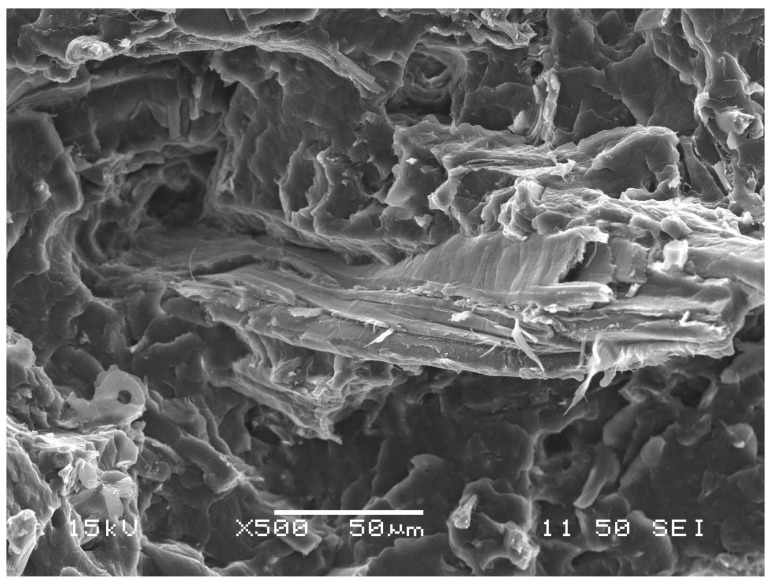
SEM micrograph recorded on the fracture surface of the hPP3/W68 composite containing 15 vol% wood flour in the presence of the MAPP2 coupling agent. Longitudinal fracture of a large wood flour particle.

Depending on their size and anisotropy, lignocellulosic fibers can break parallel to their axis as well. The longitudinal fracture of a large wood flour particle can be observed on the SEM micrograph (Figure 8) recorded on the fracture surface of a PP composite containing 15 vol% wood flour at good adhesion, i.e., in the presence of MAPP coupling agent. It is worth pointing out that in PP/wood composites debonding is the dominating deformation process at poor, while the fracture of fibers at good adhesion. Consequently, the strength of interfacial interactions determines the mechanism of the dominating local deformation process occurring around inclusions.

## 6. Correlations, Consequences

**Figure 9:**
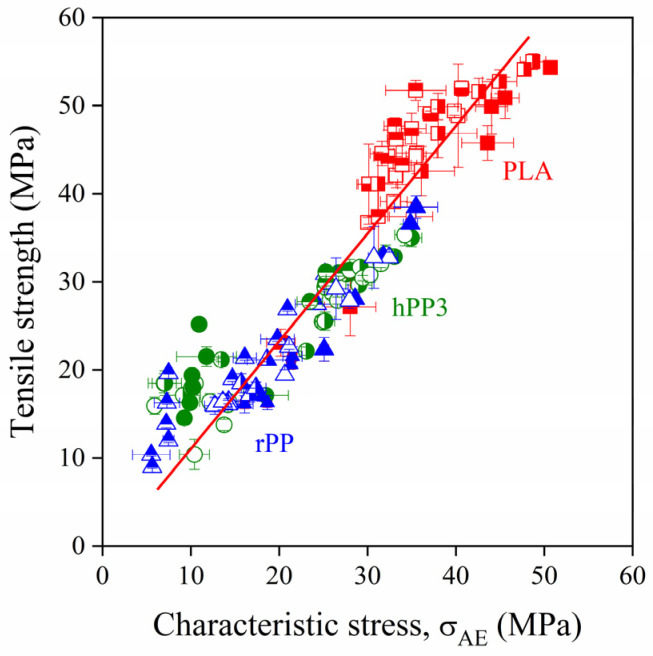
Close correlation between the initiation stress of the dominating local deformation process and the strength of lignocellulosic fiber reinforced PP and PLA composites.

As mentioned previously, local deformation processes determine the macroscopic properties of heterogeneous polymer systems. This statement is further supported by the results presented in Figure 9, in which the tensile strength of several lignocellulosic fiber reinforced PP and PLA composites is plotted against the characteristic initiation stress derived from acoustic emission results. An unambiguous correlation exists between the two quantities, confirming that the dominating local process determines the strength of the composites irrespective of its mechanism. The correlation is valid independently of the type of the reinforcement as well as the matrix polymer used in the study and it also indicates that composite properties can be improved by the proper control of the micromechanical deformation processes occurring during deformation.

**Figure 10:**
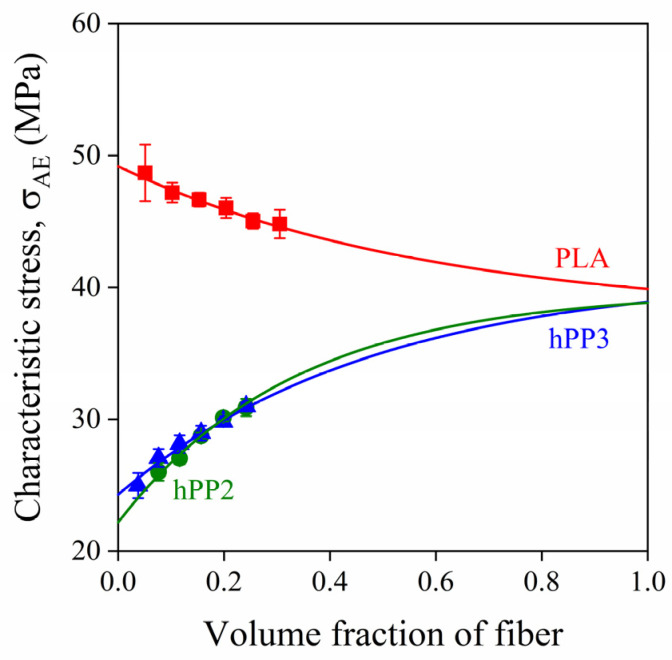
Determination of the inherent strength of sugarpalm fibers from the composition dependence of characteristic stress values derived from acoustic emission testing at good adhesion.

At good adhesion, fiber fracture is frequently the dominating deformation mechanism in lignocellulosic fiber reinforced composites. As shown by Figure 9, the initiation of the process leads to failure, thus the upper limit of reinforcement is determined by the inherent strength of these fibers. If we plot characteristic stresses determined by acoustic emission testing at good adhesion against fiber content and extrapolate them to the volume fraction of 1, the inherent strength of lignocellulosic fibers can be obtained, as shown in an earlier study [32]. The method of determination is demonstrated in Figure 10 for sugarpalm fiber-reinforced PP and PLA composites. As shown by the figure, the fitted exponential correlations converge on the same final value which is obviously the inherent strength of the fibers. We obtain very similar values for the two PP grades and PLA validating the procedure further. We have to point out that the approach can be used only if adhesion is strong between the components, and fiber fracture dominates failure [32].

**Figure 11:**
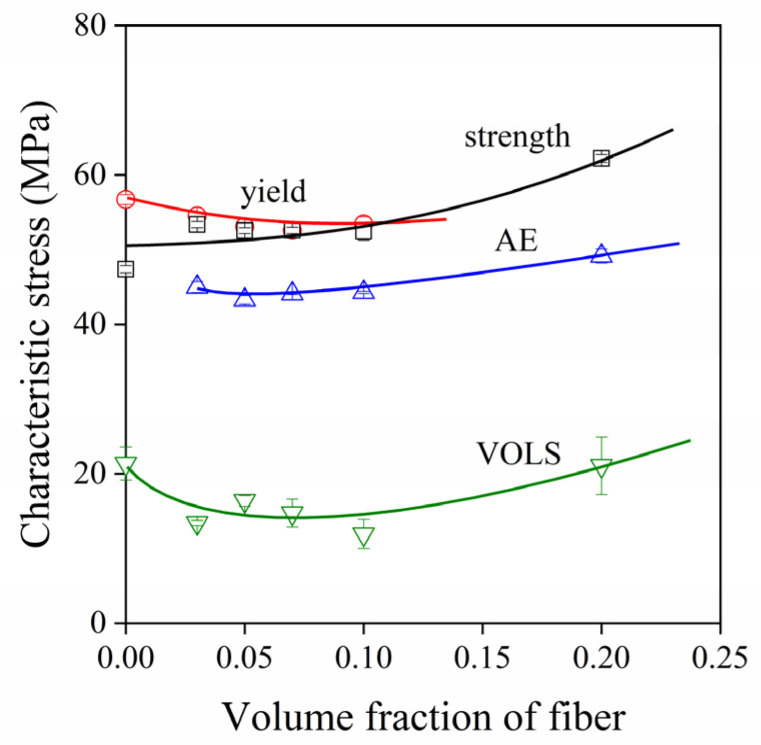
Characteristic stresses derived from various measurements plotted against fiber content for PA6/W68 composites.

Although the initiation of the dominating particle related local process determined mechanical properties in both PP and PLA composites, exceptions also exist. A good example is offered by Figure 11 in which the characteristic stresses derived from different measurements carried out on PA/wood flour composites are plotted against fiber content. Several local processes take place in the matrix and around the inclusions in these composites. Volume increase (VOLS) is caused mainly by the cavitation of the matrix, while acoustic emission signals (AE) are related to the fracture of wood flour particles. The characteristic stresses of both processes are much smaller than the tensile yield stress and the tensile strength of the composites, indicating that the initiation and occurrence of local processes do not lead to the immediate failure of the material. Accordingly, not local processes, but the characteristics of the matrix polymer determine composite properties and performance in this case.

Interfacial adhesion is claimed to be a factor of crucial importance in determining the properties of heterogeneous polymer systems [10,11,33]. The strength of interfacial interactions can be quantitatively estimated by the determination of the reversible work of adhesion. This can be calculated from the surface tension of the components and/or acid–base interactions when adhesion is created by secondary, van der Waals forces [34]. Since a good correlation exists between interfacial adhesion and several mechanical properties [35,36], surface modification techniques are often applied in polymer composites to reach the desired property profile. Functionalized polymers, maleated polyethylene (MAPE) or polypropylene (MAPP) are frequently used in both short fiber reinforced and nanocomposites to achieve proper adhesion. In glass fiber-reinforced composites aminosilane treatment is a commonly applied modification route to increase the strength of interactions. The concept of the reversible work of adhesion for the calculation of the strength of interfacial interactions cannot be used for the materials listed above, since in such cases adhesion is created by stronger, covalent/ionic bonds or the interdiffusion of the coupling agent and the matrix polymer.

Acoustic emission makes possible the quantitative estimation of the adhesion strength for composites in which interactions are created by mechanisms other than van der Waals forces, as shown in a previous paper [37]. The concept can be used only if debonding is the dominating deformation process. The approach is based on the model describing the stress needed for the initiation of debonding developed by Vörös and Pukánszky [30]. The model is expressed in the form of Equation (1)

(1) $$\sigma^{D}=-C_{1}\sigma^{T}+C_{2}{\left(\frac{EW_{AB}}{R}\right)}^{1/2}\;$$

where $\sigma^{D}\;$and $\sigma^{T}\;$are debonding and thermal stresses, respectively, E is the Young’s modulus of the matrix, $W_{AB}$ is the reversible work of adhesion and R denotes the radius of the particle. The value of constants $C_{1}$ and $C_{2}$ depend on the geometry of the debonding process and on the propagation of the crack along the surface of the particles. The model cannot be used for the prediction of adhesion strength in the form presented in Equation (1), since $C_{1}$,$C_{2}$ are unknown. If debonding stress could be determined in an appropriate way for a composite with known reversible work of adhesion, the value of $C_{1}$ and $C_{2}$ could be obtained and then applied for further calculations. Characteristic stress determined by acoustic emission, $\sigma_{AE}$, is related to the initiation of the dominating local deformation process as presented in Figure 1. If debonding dominates during deformation, $\sigma_{AE}\;$can be used as $\sigma^{D}$. 

**Figure 12:**
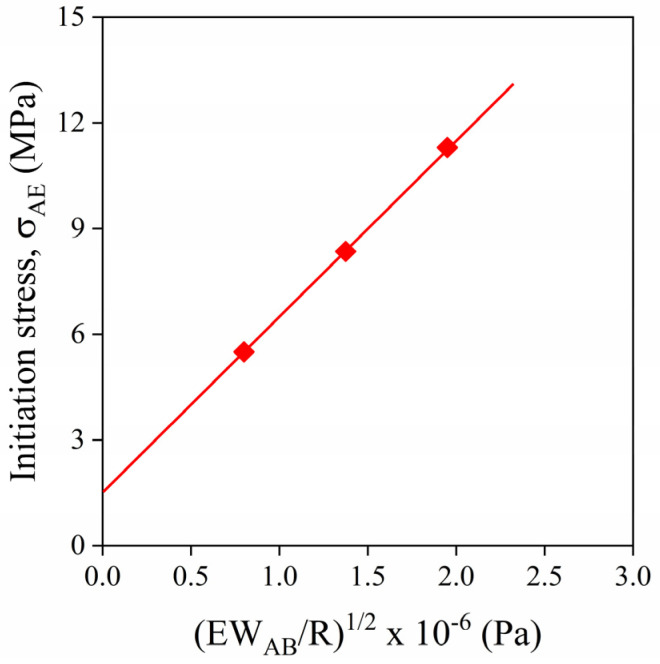
Determination of the constants $C_{1}$ and $C_{2}$ of Equation (1) from initiation stresses determined by the acoustic emission testing of hPP1/CaCO_3_ composites containing particles with different sizes. Filler content was 20 vol%.

The determination of constants $C_{1},C_{2}$ was carried out on PP/CaCO_3_ composites with different particle size distribution at 20 vol% filler content [37]. Matrix modulus of 1,5 GPa and reversible work of adhesion of 107 mJ/m^2^ for uncoated CaCO_3_ were used in the calculation. Thermal stress, $\sigma^{T}$**,** was estimated to be 10 MPa [38]. As shown by Figure 12, parameters $C_{1}$ and $C_{2}$ can be obtained as the intersection and the slope of the straight line if the initiation stress of debonding $\left(\sigma_{AE}\right)$ derived from acoustic emission measurements is plotted as a function of ${\left(\frac{EW_{AB}}{R}\right)}^{1/2}$. The goodness of the linear fit was close to 1 [37]. The constants $C_{1}$, $C_{2}\;$calculated by fitting allow us the quantitative determination of the strength of adhesion irrespectively of its mechanism for any composite in which debonding is the dominating local deformation process. As shown by Table 2, the strength of interfacial adhesion derived from the approach is very close to that calculated directly, thus verifying the proposed procedure. It is also shown that interfacial adhesion is almost 10 times larger with coupling than without it. This fact corresponds to our expectation, since MAPP can create strong ionic bond with the surface of CaCO_3_.

## 7. Conclusions

Polymers used in load-bearing applications often contain a second component, resulting in heterogeneous structure. Stress concentration develops around inclusions in such materials, which are exposed to external load thus initiating local deformation processes. Attaching a piezoelectric sensor to the specimen makes possible the detection of signals during tensile testing. The approach, called acoustic emission measurement, is an effective technique for the study of local deformation processes, since most of them are accompanied by the generation of elastic waves. The evaluation of several aspects of the results obtained in the measurement allows us to identify the deformation mechanisms with acceptable probability. The amplitude of the acoustic events, the shape of the cumulative number of signals trace, as well as characteristic deformation and stress values offer valuable information about the processes occurring in heterogeneous polymeric materials. Unfortunately, the unambiguous identification of the deformation mechanisms is not possible based on the results, but SEM micrographs help evaluation further. Supplementary volume strain measurements can be also carried out in order to identify processes, such as cavitation, which do not emit much sound. The lack of signal development may limit the use of acoustic emission testing. Small particle sizes, weak interactions, small matrix modulus and thin specimens hinder wave generation and/or propagation which may lead to the detection of only very few events. Micromechanical deformation processes strongly affect the failure characteristics of heterogeneous polymer systems. In order to improve performance, these processes must be detected and studied appropriately. For this purpose, acoustic emission testing is a very good tool with its advantages and drawbacks as well.

## Figures and Tables

**Table 1:** Composition of the various materials discussed in the paper and the corresponding figures in which their results are presented.

Related Figure	Matrix	Filler	Surface Modification ^a^	Composition (vol%)
1	hPP1	CaCO_3_	-	15
2	hPP1	CaCO_3_	-	0, 5, 10, 15, 20, 25, 30
			stearic acid	
			MAPP1	
		glass bead	-	
			aminosilane	
3	hPP1	CaCO_3_	MAPP1	0, 5, 10, 15, 20, 25, 30
4	hPP1	CaCO_3_	-	0, 5, 10, 15, 20, 25, 30
			MAPP1	
5	ionomer	lignin	-	20
6	hPP3	W68	-	15
7	hPP3	glass fiber + PVA fiber	MAPP2	15 + 15
8	hPP3	W68	MAPP2	15
9 ^b^	hPP3, rPP, PLA	CC23, W35, W54, W68, W126	-	0, 5, 10, 15, 20, 25, 30
10 ^b^	hPP2, hPP3, PLA	sugarpalm fiber	MAPP2	0, 5, 10, 15, 20, 25, 30
11	PA6	W68	-	0, 5, 10, 15, 20
12	hPP1	CaCO_3_	-	20

^a^ The amount of maleated polymer was 10% calculated for the weight of the filler and added on the expense of the matrix polymer in all composites; ^b^ All combination of the listed materials were prepared in the project.

**Table 2:** Strengths of interfacial adhesion determined by the proposed approach in PP/CaCO_3_ composites at various surface modifications.

Filler	Surface Modification	Adhesion Strength (mJ/m^2^)	
		Direct Calculation	Using the Approach
CaCO_3_	-	105	99
	stearic acid	65	51
	MAPP		861

## Data Availability

The raw/processed data required to reproduce these findings cannot be shared at this time due to legal or ethical reasons.
